# Medical education: an Italian contribution to the discussion on global health education

**DOI:** 10.1186/s12992-020-00561-8

**Published:** 2020-04-08

**Authors:** Giulia Civitelli, Gianfranco Tarsitani, Alessandro Rinaldi, Maurizio Marceca

**Affiliations:** 1 Public Health and Infectious Diseases Department, Sapienza University of Rome, Piazzale Aldo Moro 5, 00185 Rome, Italy; 2 Italian Network for Global Health Education (INGHE), Rome, Italy; 3 Italian Society of Migration Medicine (SIMM – Società Italiana di Medicina delle Migrazioni), Rome, Italy; 4 Caritas Medical Area, Rome, Italy; 5 Italian Society of Medical Education (SIPEM – Società Italiana di Pedagogia Medica), Rome, Italy

**Keywords:** Global health, Medical education, Medical ethics, Determinants of health, Inequities in health, University’s third mission

## Abstract

**Background:**

In Italy an important contribution to the spread of global health education (GHE) grew from the establishment and work of the Italian Network for Global Health Education (INGHE). INGHE gave a national shared definition of global health (GH), grounded in the theory of determinants of health, inspired by a vision of social justice, and committed to reduce health inequities. The aim of this article is to share with the international community INGHE’s point of view on Medical Education.

**Methods:**

To express its view of medical education at the national level, INGHE established a dedicated commission, which elaborated a first draft of the document and then shared and discussed it with all other members.

**Results:**

INGHE elaborated a paper where it explained the need to change medical education in order to prepare future health professionals for the challenges of the globalized and unequal world. In this article the authors summarize the experience of INGHE and share with the international community its document.

**Conclusions:**

The authors believe it is necessary now, more than ever, to insert this new approach to health at social and academic levels. Students should play a fundamental role in the spread of GHE, and activities related with GHE could be considered an important part of the third mission of universities to promote social justice.

## Background

The current social and political situation at the international and domestic Italian levels stimulates the authors of this paper to share with the international community the experience of the Italian Network for Global Health Education (INGHE) and the reflections produced by this network. The originality of this article relies on the presentation to the international community of the document “Change Medical Education”, which contains INGHE’s perspective on Medical Education and Global Health Education (GHE).

The necessary introduction to this article is the determined belief that there could and should not be any neutrality in the academic and scientific field, and especially in the medical field [1]. As health professionals are concerned about the health and life of the people they serve, they cannot stay neutral in front of political choices and social situations [2, 3]. These considerations should have consequences on how future doctors and health professionals are educated at universities. As the Lancet Commission noted, the education of health professionals needs to be transformed [4]. In the past few decades, the spread of global health education in Europe and beyond has been extensively documented [5–20], but up until now the Italian perspective has not been well disseminated outside of Italy. For this reason, the authors present the work of INGHE of the past years and share with the scientific community some final considerations for future practices.

INGHE is a national network formed by academic institutions, scientific societies, non-governmental organizations, associations, groups and individuals engaged in teaching global health (GH) in universities and in civil society. The establishment of INGHE was the outcome of a process that started in 2007, stimulated by the European project ‘*Equal opportunities for health: action for development’*, coordinated by the Italian NGO Doctors with Africa CUAMM, with the active participation of medical students in Italy. Project work brought together students, young doctors and researchers, lecturers, health professionals and non-governmental organizations to exchange ideas on issues of common interest and experiences including: international health and the inequalities existing between and within countries; the links between market, globalization and health; and social determinants of health. The will to continue this exchange and cooperation beyond the life of the project and the desire to involve other national players, led to the creation of INGHE in 2010 [21, 22].

INGHE’s general objective was to contribute to improving the health of the population and to reduce existing inequalities both within each country and between various countries, by improving health provider knowledge, attitudes and practices.

The specific objectives of INGHE since 2010 were:

- to contribute to the drafting, discussion and spread of the GH approach;
- to promote GH teaching both at the academic level (in health-oriented degree courses and medical residency schools) and the professional level (courses within the framework of continuing education in medicine programs);
- to promote time and space for exchange and dialogue on GH with other disciplines and with institutions, groups, associations and networks in Europe;
- to promote a public debate on GH issues and to raise awareness among institutions about bridging the gap between evidence and practice.

Since 2010, various targets have been met. Firstly, INGHE’s members agreed to a shared definition of GH.

*In our view, GH is meant to be a new paradigm for health* [23] *and health care, grounded in the theory of health determinants. Such an approach, based on the principles stated in the Alma Ata declaration* [24] *and backed by broad evidence,* [25] *can be applied to disease prevention, diagnosis and treatment, as well as to health promotion, for both individuals and populations.*

*The main focus of GH concerns the health status and the real needs of the world’s population, as well as its socio-economic, political, demographic, juridical and environmental determinants, as well as the relationship between globalisation and health in terms of equity, human rights, sustainability and international diplomacy. Due to the complexity of such issues, the GH approach is necessarily a trans-disciplinary and multimethod one, built on the contribution of natural, biomedical and social sciences and the humanities.*

*Adopting a transnational view, GH points out health inequalities both within and among countries, framing them also through the lens of social justice. GH is not merely an academic field: fostering an ethics of social accountability for institutions, professionals and individuals involved, it encompasses the fields of research, practice and education, aiming to produce change in the community and in the whole society, and bringing evidence into practice thus reducing the know-do gap* [26]*.*

Starting from this definition, INGHE defined the main objectives and contents of a GH course, the didactic methodologies that should be used, and the instruments for the evaluation of courses.^1^ Two editions of a national survey of GH courses were carried out. Reflections were made on the importance of participatory experiences in the community [27].

At the end of 2014 the Italian Medical Association (FNOMCeO) began a reflection process concerning medical education. The Permanent Conference of the Presidents of Degree Courses in Medicine also gave its contribution. Informed with its recent pertinent insights, INGHE decided to elaborate its own reflections with a consensus process. The objective was to produce a paper which summarized the point of view of INGHE on medical education and GHE and to share it with other actors in the country, as an opportunity to continue the debate inside Italy. Subsequently believing it could offer an important contribution to discussions regarding GHE, INGHE intends to share its conclusions with the international community.

## Methods

A dedicated commission was established from among the members of INGHE. The members of the commission were young residents in Hygiene and Preventive Medicine (Public Health) supervised by two professors from universities in Rome (Sapienza University of Rome) and Bologna (Alma Mater University). The first phase consisted of a broad-based literature review concerning “global health education” and “medical education”. A deep analysis of two official documents of the Italian Medical Association and The Permanent Conference of the Presidents of Degree Courses in Medicine was conducted, as they provided a mainstream, external perspective. The second phase was characterized by brainstorming and discussion among the members of the commission, to identify the principal points that should be included in the document. The third phase resulted in the elaboration of the first draft of the paper. Finally, the draft was discussed and reviewed by all members of INGHE. No standardized methodologies have been used to reach the consensus. The draft was shared among the members of INGHE, all of whom were able to read and analyse it independently prior to the group meeting. Consensus was reached after discussions in the plenary session. In March 2015 the final paper was disseminated in Italy [28].

## Results

We present INGHE’s final outcome.

**Table:** 

***CHANGE MEDICAL EDUCATION*** ***The contribution of the Italian Network for Global Health Education*** ***Introduction*** *Are medical schools able to educate health professionals who can give an answer to the health needs of people and the communities they will work for? How do medical schools answer to the challenges in a time of globalization and complexity? How do they deal with the issue of social responsibility and social justice? The Italian Network for Global Health Education (INGHE) believes that these questions should be taken into consideration and it expresses in this paper its contribution to the debate regarding medical education.* *In summary, in the opinion of the INGHE:* - *Every action and decision taken in the medical field is not ethically neutral. Medicine implies intrinsic ethical aspects and it should be studied and taught starting from these ethical components.* - *The paradigm of complexity, which is typical of our time, provokes the recognition of the intrinsic limits of every human practice, including medicine. It is necessary to create moments of dialogue and debate between fields of knowledge, disciplines and professions.* - *Within the matrix of education, it is necessary to promote the development of critical thinking and to encourage ethical choices. This is possible by stimulating moral reflection with the contribution of different disciplines. Educational experiences outside universities, in the places where people live and work, could be important in this direction.* - *It is important to reduce overspecialization in order to give space to a “new generalism” whereby a wider approach can consider health and disease inside peoples’ life continuum.* - *It is necessary to make a call to social responsibility for current and future health professionals. Social responsibility should inform and influence crisis situations, social injustice, exclusion caused by globalization. This responsibility isn’t decided “*a priori*”/ from the beginning; it should be actively sought on a personal level and in debates with every person who wants to give their contribution.* ***From global health to medical and health professionals’ education*** The Italian Network for Global Health Education (INGHE) is a national network formed by academic institutions, scientific societies, non-governmental organizations, associations, groups and individuals engaged in teaching global health, at universities and in civil society. The establishment of INGHE was the outcome of a process that started in 2007, stimulated by the European project, “Equal opportunities for health” and the active participation of medical students in Italy. Similarly, the Secretariat of the Italian Medical Students (SISM) has been an important active part of INGHE since the beginning. INGHE is concerned about the actual organization of medical schools and aims to open a national debate on medical education, global health education and “health education” in a wider sense. As members of INGHE we believe that teaching global health means introducing a new way to think and act concerning health while “aiming to produce change in the community and in the whole society, and bringing evidence into practice, thus reducing the know-do gap”. This is the reason why INGHE, which began its work considering only medical education, recognises the necessity to take into consideration educational paths of the diverse professions involved in the safeguarding and promotion of health. According to INGHE it is necessary a reform not only medical schools, but also educational paradigms. ***Medicine as an ethical practice*** Medical education’s reform should start from the awareness that to think about medicine only as a science or a scientific activity is a substantial mistake. Medicine, as a practice, implies actions that express different meanings and purposes. Ethical aspects should be considered part of it. Every decision and action taken in this field is not neutral, and it is not possible to disregard ethical considerations. This means that medicine should be studied and taught starting from an ethical perspective. This approach should not be limited to reflections around doctor-patient relationships, but it should be extended also, for example, to relationships between medicine and other fields of knowledge. This could help to identify deficiencies and weak points where it is necessary to work. The real question is: are current medical schools able to educate future health workers to have sufficient ethical and scientific knowledge needed to inform citizens and professionals while considering the complex systems in which they live every day? ***For a new generalism*** In a time characterized by the exponential increase of scientific and technical knowledge, academic curricula are becoming systems of rote learning in order to pass exams. Moreover, the request of hyper-specialization provokes an educational blackmail that obliges graduates to continue studies waiting for a place in residency. In Italy this time between graduation and residency is in limbo, a state of uncertainty. As knowledge becomes ever more hyper-specialized and fragmented, doctors risk transforming themselves into competent technicians. This reductionist organization, based on superficial factual knowledge, is the consequence of the fracture between science and ethical acts, typical of positivist culture. A fragmented knowledge base is not able to produce future health professionals who are able to answers health’s needs of people and communities they are going to serve. Hyper-specialization provokes a growing estrangement of doctors from places of people’s everyday life. Medical education takes place mostly inside lecture halls and hospitals, impeding future doctors’ from an awareness of factors that influence health in different social contexts. Ageing and the increase of non-communicable diseases require a wider approach, which should give centrality to aspects of prevention, health promotion, primary health care and health and social care integration [29]. For this reason, we believe that it is important to reduce hyper-specialization to give space to a new generalism that considers health and disease inside people’s life continuum [30]. ***The necessity of wise choices*** The vertiginous increase of diagnostic and therapeutic possibilities and the social construction of the power of medicine have fed an ingenuous trust in the ability of medical professions to free man from pain, suffering and death. The growing pressure of bio-medical and pharmaceutical industries contributes to a progressive medicalization of every aspect of human life (i.e. the phenomenon of disease mongering [31], and a consequent induction of false needs). Expectations of people who access health services are growing, as well as health expenditure, and there is an increasing inappropriateness of services. The context of economic crisis and the partial shortage of resources warrant wise choices for the allocation of expenses. It is important that these choices go in the direction of equity and universal access to health care. A utilitarian approach that exclusively follows economic criteria needs to be rejected. Starting from education, it is important to reflect upon the concept of limits, and upon the necessity of wise and ethically founded choices, oriented to avoid squandering and fighting against both corruption and conflicts of interest. ***Doctors’ social responsibility*** There are growing scientific evidence [25] of the unequal distribution of diseases between and within countries, in relation to social divisions (described with different kinds of indicators of socio-economic positions). Health inequities are related to social determinants of health; in order to reduce them, it is important to act on all the factors (not simply biologic ones) that could influence an individual or a community’s health. This does not have to give medicine an excessive task. It is necessary to call future doctors to a responsibility beyond the doctor-patient relationship, and moves towards a responsibility that encompasses considerations of the greater society [32]. Health professionals are able to recognise and scientifically prove the real consequences of political and economic systems on peoples’ lives and health. Therefore, they cannot remain neutral in front of these inequities. With this in mind, doctors and future health professionals should enter into dialogue with different sectors of society and with disciplines that work for the common good. This is not a technical or facultative aspect, but rather an ethical imperative. ***Change medical education: a social matter*** These are only a few examples that show the necessity to insert wider knowledge and ethical reflections within medical education [4]. In other words, medical education should provide the instruments to develop critical thinking and to face the complexities of reality, and promote experiences that encourage free and responsible answers to challenges and problems of a globalised world. These challenges and problems, touchable and visible among the poor and excluded, provoke the need to recognise the limits of every human act related to one’s own person, role or education. That’s why every act, grounded on a real and critical choice and ethical values, should recognise the importance of collaboration among different professions and disciplines. Reflections and practical experiences related with the concept of solidarity, responsibility, justice, equity, limits, and cooperative thinking should be considered an important part of medical education. It is essential to involve medical students so that they can become active protagonists of their education. It is important to give value to other discipline’s points of view, so that they can help to analyse the context of crises, not only economic but also cultural, ethical and anthropological, in which medical faculties (and the whole academic world) are involved. This in a necessity related with the limits of medicine, limits that are more evident in complex systems where health professionals are called to work. Now is the moment to create the basis for a new pedagogy of health and this is a cultural, organizational, ethical, civil and professional endeavour. Thus, not only future workers, but first and foremost citizens, for a society where equity and social justice should become fully-fledged instruments of health.	

## Discussion

The diffusion of the document “Change medical education” [28] stimulated a debate in Italy for the first year. After that, for various reasons, discussions related to GHE in Italy decreased.

Even if it is true that no standardized methodologies have been used to elaborate the document, the authors believe that it could offer a stimulating contribution to the debate on Medical Education both at national and international level. It is important to revive once again the debate and to work so that universities can formally recognise the importance of this approach and those arguments for the education of future health professionals.

The cultural debate related with global health has led to the change of the article n° 5 of the Italian Code of Medical Ethics. Currently the article affirms that “the doctor, considering the context of life, work, level of education and social equity as fundamental determinants of individual and collective health, collaborates to the fulfilments of educational, preventive and other kind of policies for the contrast of health inequities and promote the adoption of healthy lifestyle giving information regarding the main risk factors” [33]. At the end of the article there is also a reference to the contribution that doctors should give for a “liveable ecosystem also for the future generation” [33].

In 2018 a working group was established in the context of the Italian Medical Association, related to “Global Health, Inequities in Health and International Cooperation”. One of the authors of this article is part of this working group. The group was motivated to stay in contact with universities and medical faculties in Italy to spread global health education in the academic world.

It is particularly important to speak about the social responsibility of doctors and future health professional at this time when themes and phenomena such as migration and climate change are being discussed. The social responsibility of health professionals is undeniable, especially in consideration of their positions at the fore of situations influenced by poverty, marginalization and health inequalities. Doctors should have an instrumental role in leading efforts to ensure the wellbeing of marginalized persons, and all those who suffer the consequences of inequities. The phenomenon of migration is, for example, a good “case study” for a GH approach. It is the most present challenge in western societies, especially in southern Europe and Italy, and must be addressed directly. Currently, anti-immigrant positions seem to prevail in Italy. Present and future health professionals must take the position to defend the right to health of every human being, including undocumented migrants and, in general, all marginalized people. Students must become aware of the importance of the Public National Health System; they should know that undocumented migrants in Italy can access healthcare and the procedures available to do this. Moreover, students should take positions against new discriminatory laws concerning migration, because they are pathogenic for people and for the society. Discriminatory laws could be a good example of legal determinants of health [34].

Concerning climate change, and ecological issues in general, medical and health profession students should become aware of the consequences of climate change on the health of the people. They should study the causes of climate change and should promote the social change regarding these questions. These crucial debates of our time, as underlined in international documents, such as *Sustainable Developments Goals - SDGs* [35] and influential voices, such as Pope Francis’s *Laudato Si′* [36].

The debate on Medical Education and the need to deeply reform Medical Education globally is a very important one. Especially in light of the global effort to align to SDGs and to revitalize a universal and comprehensive approach to health (as SGD3 states: “ Ensure healthy lives and promote well-being for all at all ages”), it is important to reassess the knowledge and skills currently absent from the Medical curriculum. It is hoped that reading and sharing the document produced by INGHE will encourage a reflection on medical and health professionals’ education so that future doctors and health workers could become committed to social justice for a better health for all.

## Conclusions

In the context of the globalized world, it is necessary to rethink Medical Education; GHE appears to be essential to prepare future health professionals to face present and future challenges. Since 2010 the Italian Network of Global Health Education (INGHE) has worked to find shared definitions, main objectives, didactic methods and evaluation forms of GH courses. INGHE elaborated an important document in 2015 and its contents have now been translated and published in this paper.

Considering the increasing influence of globalization on societies, people and diseases, it is fundamental to continue the debate regarding GHE at national and international levels. The authors believe that INGHE and Italian professionals could give an important contribution to this debate.

To include GH related issues and approaches in medical training, future pathways of action may comprehend proposals for more GH courses and masters at universities, and the promotion of academic research in this field. Coherently with a “GH approach”, in order to avoid promoting the dynamics of power within societies, it is important to involve students from the outset, so that they can actively participate in the organization of the courses. It is likewise important to give to students and professionals, regardless of their socio-economic levels, the opportunity to access these kinds of studies. There is a need to address the prohibitive costs of academic courses, which obstruct wider accessibility.

The authors believe that GHE should be an important part of the “third mission” in universities. This expression is often related to the relationship between universities and industry and markets. It is not enough underlined the active role that universities could have in social engagement, concerning for example inequities and social problems [37].

It is time for all stakeholders (student ‘associations, universities, scientific societies, medical associations, non-governmental organizations, single doctors and people) to engage once again in the diffusion of the social determinants of health with a view to tackle health iniquities and promote social justice.

## Data Availability

Not applicable.

## Abbreviations

GH: Global Health
GHE: Global Health Education
INGHE: Italian Network for Global Health Education

## Footnotes

1 Documents produced and activities promoted by INGHE are available online: http://educationglobalhealth.eu/en/
